# 

**Published:** 1916-09

**Authors:** 


					﻿Vol. XXXII
SEPTEMBER, 1916
No. 3
TEXAS
Medical Jc^nal
ESTABLISHED JULY, 1885
PUBLISHED MONTHLY AT AUSTIN, TEXAS, BY
MRS. F. E* DANIEL, dr •' Publisher and Managing Editor
Subscription, 81.00 a Year, in ^dYance. «	- - *	* Single Copies, 10 Cents
Entered at the Postoffice at Austin, Texas, as second class mail matter.
0£
<
U
>
Q
Z
0
1
fl
				

